# Ambient Fine Particulate Matter Suppresses *In Vivo* Proliferation of Bone Marrow Stem Cells through Reactive Oxygen Species Formation

**DOI:** 10.1371/journal.pone.0127309

**Published:** 2015-06-09

**Authors:** Yuqi Cui, Fengpeng Jia, Jianfeng He, Xiaoyun Xie, Zhihong Li, Minghuan Fu, Hong Hao, Ying Liu, Dylan Z. Liu, Peter J. Cowan, Hua Zhu, Qinghua Sun, Zhenguo Liu

**Affiliations:** 1 Dorothy M. Davis Heart and Lung Research Institute, Division of Cardiovascular Medicine, The Ohio State University, Columbus, OH, United States of America; 2 Department of Cardiology, Shandong Provincial Hospital, Shandong University, 324 Jing 5 road, Jinan, Shandong 250021, P.R. China; 3 Department of Surgery, Wexner Medical Center, The Ohio State University, Columbus, OH, United States of America; 4 Department of Medicine, University of Melbourne, St. Vincent’s Hospital, Melbourne, Australia; 5 Department of Cardiovascular Medicine, the First Affiliated Hospital,Chongqing Medical University, Chongqing 400016, China; University of Cincinnati, College of Medicine, UNITED STATES

## Abstract

**Aims:**

Some environmental insults, such as fine particulate matter (PM) exposure, significantly impair the function of stem cells. However, it is unknown if PM exposure could affect the population of bone marrow stem cells (BMSCs). The present study was to investigate the effects of PM on BMSCs population and related mechanism(s).

**Main Metheods:**

PM was intranasally distilled into male C57BL/6 mice for one month. Flow cytometry with antibodies for BMSCs, Annexin V and BrdU ware used to determine the number of BMSCs and the levels of their apoptosis and proliferation *in vivo*. Phosphorylated Akt (P-Akt) level was determined in the BM cells with western blotting. Intracellular reactive oxygen species (ROS) formation was quantified using flow cytometry analysis. To determine the role of PM-induced ROS in BMSCs population, proliferation, and apotosis, experiments were repeated using N-acetylcysteine (NAC)-treated wild type mice or a triple transgenic mouse line with overexpression of antioxidant network (AON) composed of superoxide dismutase (SOD)1, SOD3, and glutathione peroxidase-1 with decreased *in vivo* ROS production.

**Key Findings:**

PM treatment significantly reduced BMSCs population in association with increased ROS formation, decreased P-Akt level, and inhibition of proliferation of BMSCs without induction of apoptosis. NAC treatment or AON overexpression with reduced ROS formation effectively prevented PM-induced reduction of BMSCs population and proliferation with partial recovery of P-Akt level.

**Significance:**

PM exposure significantly decreased the population of BMSCs due to diminished proliferation via ROS-mediated mechanism (could be partially via inhibition of Akt signaling).

## Introduction

A recent Global Burden of Disease Study suggested that the ambient fine particulate matter (PM) PM is responsible for 3.2 million deaths per year and 76 million years of healthy life lost [1]. The majority of mortality following PM exposure has been shown to be related to cardiovascular diseases [1]. Different sources of PM contain different components. The composition of PM is a mixture of various particles including metals, crustal material and bio-aerosols [2, 3]. It has been reported that PM exposure is able to produce many deleterious effects on cardiovascular system such as vascular dysfunction, reduced heart rate variability and enhanced coagulation-thrombosis potential [2, 4]. Long-term exposure of PM accelerated the process of atherosclerosis and vascular inflammation in apolipoprotein E^-/-^ mice with high fat diet [5].

Endothelial dysfunction or injury is considered one of the major factors that contribute to the development of atherosclerosis and coronary heart disease [6, 7]. Bone marrow-derived endothelial progenitor cells (EPCs) play a critical role in vascular re-endothelialization, angiogenesis, and prevention of neointima formation after vascular injury [8–11]. The number and function of EPCs are significantly decreased in the animals exposed to PM [12, 13]. The mechanism(s) for PM exposure-induced impairment of EPCs is not fully understood. Bone marrow (BM) is a major source of EPCs [10]. Therefore, the number and function of EPCs could be intimately associated with BM stem cells (BMSCs) in the BM. It could be possible that PM exposure led to decreased number and function of BMSCs, thus resulting in (at least partially) impaired EPCs number and function. Indeed, it has been reported that a number of deleterious effects on the BM cells and BMSCs have been observed from cigarette smoking (CS) and other environmental insults [14–17].

Exposure to PM leads to increased production of reactive oxygen species (ROS) and oxidative stress [18–21]. The present study was designed to test the hypothesis that increased ROS formation could mediate the effect of PM on the population of BMSCs. We first demonstrated that PM indeed significantly decreased the BMSCs population as defined as lineage negative/Sca-1 positive (LS) and Lineage negative CD133 positive (Lin^-^/CD133^+^) cells in the BM in association with impaired pro-survival Akt signaling and reduced proliferation of BMSCs without induction of apoptosis. To further test the hypothesis, ROS production was blocked by using either antioxidant N-acetylcysteine (**NAC**) or a transgenic mouse model (**TG**) with concomitant overexpression of an antioxidant network (**AON**) of human copper/zinc superoxide dismutase (SOD)1, extracellular SOD3, and glutathione peroxidase (Gpx-1) with decreased ROS formation. We observed that NAC treatment or AON overexpression could partially reverse PM induced inhibition of P-Akt expression and effectively rescued the reduction of BMSCs proliferation by PM. Taken together, our data demonstrated that PM-mediated ROS production was indeed a major mechanism for decreased BMSCs population due to impaired proliferation of BMSCs.

## Materials and Methods

### PM exposure and animal model

All the animal experiments were performed in accordance with the Guidelines of the Animal Care Committee of the Ohio State University Medical Center, Columbus, Ohio, USA. The experimental protocols for the present study were reviewed and approved by the Animal Care Committee of the Ohio State University Medical Center. PM<4μm (Standard Reference Materials 2786) was purchased from The National Institute of Standards and Technology (NIST), which has a mean particle diameter of 2.8 μm and the major components including polycyclic aromatic hydrocarbons (PAHs), nitro-substituted PAHs (nitro-PAHs), polybrominated diphenyl ether (PBDE) congeners, hexabromocyclododecane (HBCD) isomers, sugars, polychlorinated dibenzo-*p*-dioxin (PCDD) and dibenzofuran (PCDF) congeners, inorganic constituents, and particle-size characteristics in atmospheric particulate material and similar matrices [22]. It was dispersed in solution by ultrasonication in endotoxin-free PBS for 30 min at a concentration of 0.5 μg/μl [23, 24]. Each mouse was treated with10μg PM three times per week for 1 month via intranasal instillation[25]. Endotoxin-free PBS was used as control. Wild-type (**WT**) male C57 BL/6 mice (6–8 weeks old) were purchased from Jackson Lab (Maine, USA). To evaluate the role of ROS formation induced by PM, the mice were pre-treated with NAC (1mg/ml in the drink water) for 24 hours prior to PM exposure. To further evaluate the role of ROS production in mediating the effects of PM, a TG mouse model (was kindly provided by Dr. Peter J Cowan, Department of Medicine, University of Melbourne, St. Vincent’s Hospital, Melbourne, Australia) with concomitant global overexpression of AON with decreased ROS production (6–8 weeks old, male) were used to repeat the experiment. The generation of TG mouse that has been backcrossed at least 10 generations onto the C57 BL/6 background was described in detail previously [26]. The AON enzyme overexpression level and their activities were also determined recently [27], and confirmed in our lab [28]. The littermate WT male C57BL6 mice were used as the control.

### Determination of total and phosphorylated Akt

Mouse BM cells were collected after 1 month of exposure to PM. The protein was extracted for Western Blot analysis. The primary antibody (4060) and secondary antibody (7074) were purchased from Cell Signaling (Danvers, MA, USA) and incubated with the protein preparations according to manufacturer’s recommendation. The level of total Akt (T-Akt), phosphorylated Akt (P-Akt) and β-actin was quantified as mean ± SD by using Image J software. The P-Akt was normalized with T-Akt and the T-Akt was further normalized with β-actin.

### Flow cytometry analysis for cell proliferation, apoptosis, intracellular ROS formation and BMSCs population

After exposure of mice to PM or PBS for 1 month, mouse BM was collected and the red blood cells (RBC) were eliminated with RBS lysis as described [29]. For *in vivo* BMSCs population and BMSCs proliferation analysis, mice were injected (i.p.) with 1 mg BrdU 12h before analysis[30]. After staining with Lineage cocktail (components include anti-mouse CD3, clone 17A2; anti-mouse Ly-6G/Ly-6C, clone RB6-8C5; anti-mouse CD11b, clone M1/70; anti-mouse CD45R/B220,clone RA3-6B2; anti-mouse TER-119/Erythroid cells, clone Ter-119) Pacific Blue, Sca-1 PE-Cy5, and CD133 PE (all antibodies were purchased from Biologend, San Diego, CA), cells were permeabilized and stained with anti-BrdU FITC using the BrdU Flow Kit according to manufacture’s instruction (559619, Becton Dickinson and Company BD Biosciences, San Jose, CA). For the BMSCs population analysis, the BM Lineage negative/Sca-1 positive (LS) and Lineage negative CD133 positive (Lin^-^/CD133^+^) cell population were carefully compensated (each cell population percentile was further confirmed with single antibody staining) and determined using flow cytometry as described [31]. The BMSCs apoptotic rate was determined with FACS using apoptosis kit from BD Pharmingen (CA, USA). The early apoptotic cells were defined as Annexin V FITC positive cells, while the late apoptotic cells was defined as Annexin V FITC and propidium iodide (PI) double positive cells as described [32]. The level of intracellular ROS formation in BM cells was determined using the ROS Detection Reagents-FITC (Invitrogen) as described [29]. The cells were incubated with the reagent for 10 min at 37°C. The labeled cells were washed twice with PBS. and then suspended in warm PBS for analysis using flow cytometry. The fluorescence-positive cells were quantitatively evaluated using an LSRII (BD Bioscience, CA, USA) at the wavelength of 525nm as described [29].

### Statistical Analysis

All the data were presented as means ± standard deviation (SD), and statistically analyzed using unpaired Student t-test (two-sided) for two groups of data or one way ANOVA (analysis of variance) (PRISM Version 4.0; GraphPad Software, Inc., San Diego, CA) followed by post hoc conservative Tukey’s test for three or more groups of data to minimize type I error as appropriate. The differences were considered statistically significant when a two-tailed p < 0.05.

## Result

### PM treatment decreased BMSCs number without induction of apoptosis

**Fig 1:**
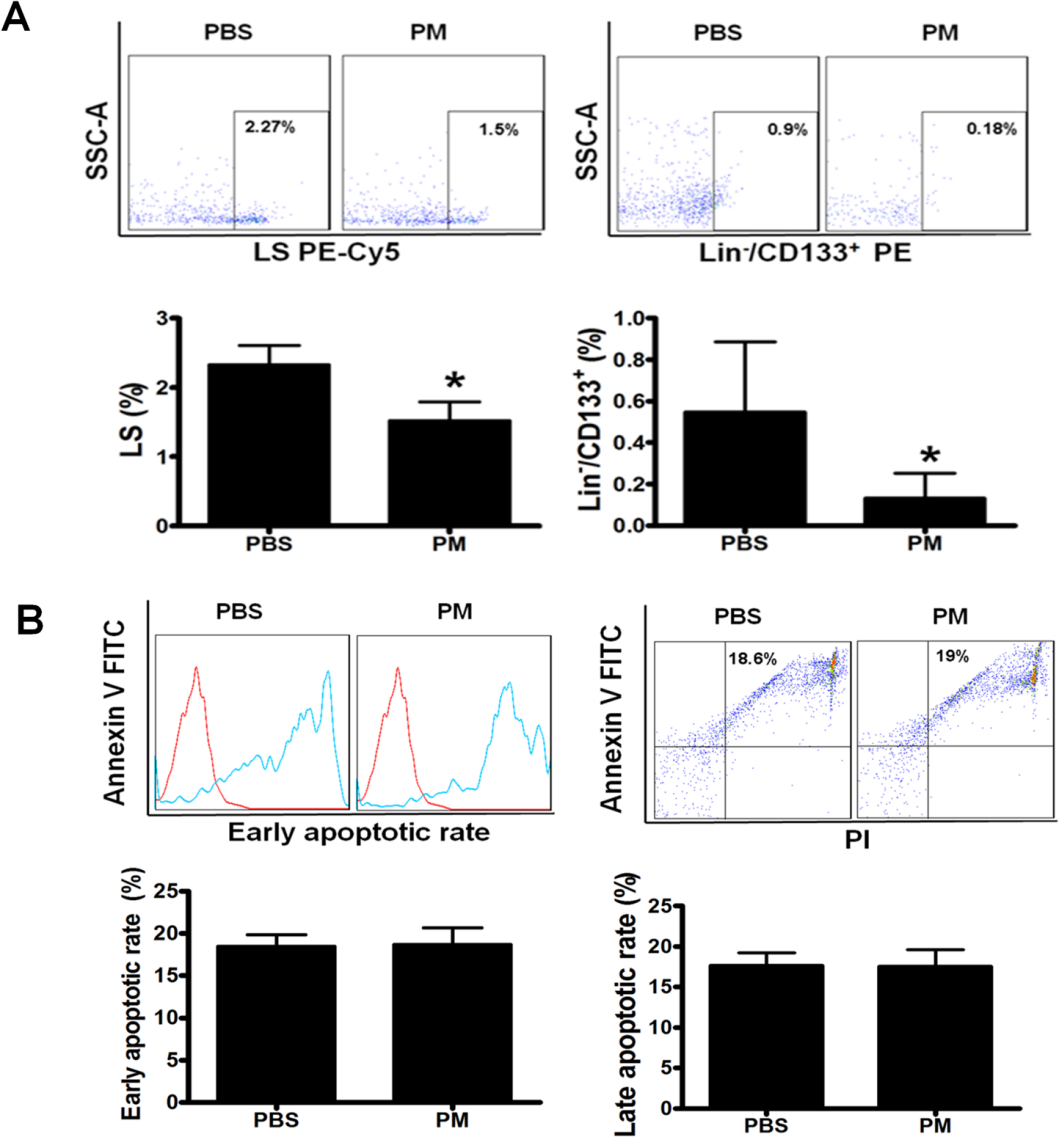
PM treatment decreased murine BMSCs level without induction of apoptosis. After C57BL/6 mice were exposed to PM or PBS through intranasal distillation for 1 month, the BM cells were collected after treating with red blood cell lysis and stained with Lineage cocktail Pacific Blue, Sca-1 PE-Cy5 and CD133 PE antibody for flow-cytometry analysis for BMSCs as defined as lineage negative Sca-1 positive (LS) and Lineage negative CD133 positive (Lin^-^/CD133^+^) cells. The BMSCs number was significantly decreased in C57BL/6 mice with PM exposure compared to the PBS control (A). Annexin V and PI were used to incubate the cells for apoptosis analysis. Both early and late apoptotic rates of cells in the mice with PM exposure were similar to the control PBS group (B). PBS: C57BL/6 mice with PBS treatment; PM: C57BL/6 mice with PM exposure. * PM vs PBS, P<0.01, n = 8.

BM cells were collected for BMSCs population analysis after PM exposure. Flow cytometry analysis showed that PM exposure significantly decreased the populations of LS and Lin^-^/CD133^+^ cells by 35% and 76%, respectively, as compared to the control group (**Fig 1A**). To determine if the decreased cell population by PM exposure could be due to increased apoptosis, we evaluated the level of apoptosis of BMSCs. As shown in **Fig 1B**, neither early nor late apoptotic rate of BMSCs with PM treatment was changed as compared to PBS control. Thus, our data suggested the mechanism for detrimental effects of PM on BMSCs might be through an apoptosis independent pathway.

### PM exposure suppressed *in vivo* BMSCs proliferation in association with decreased Akt phosphorylation

**Fig 2:**
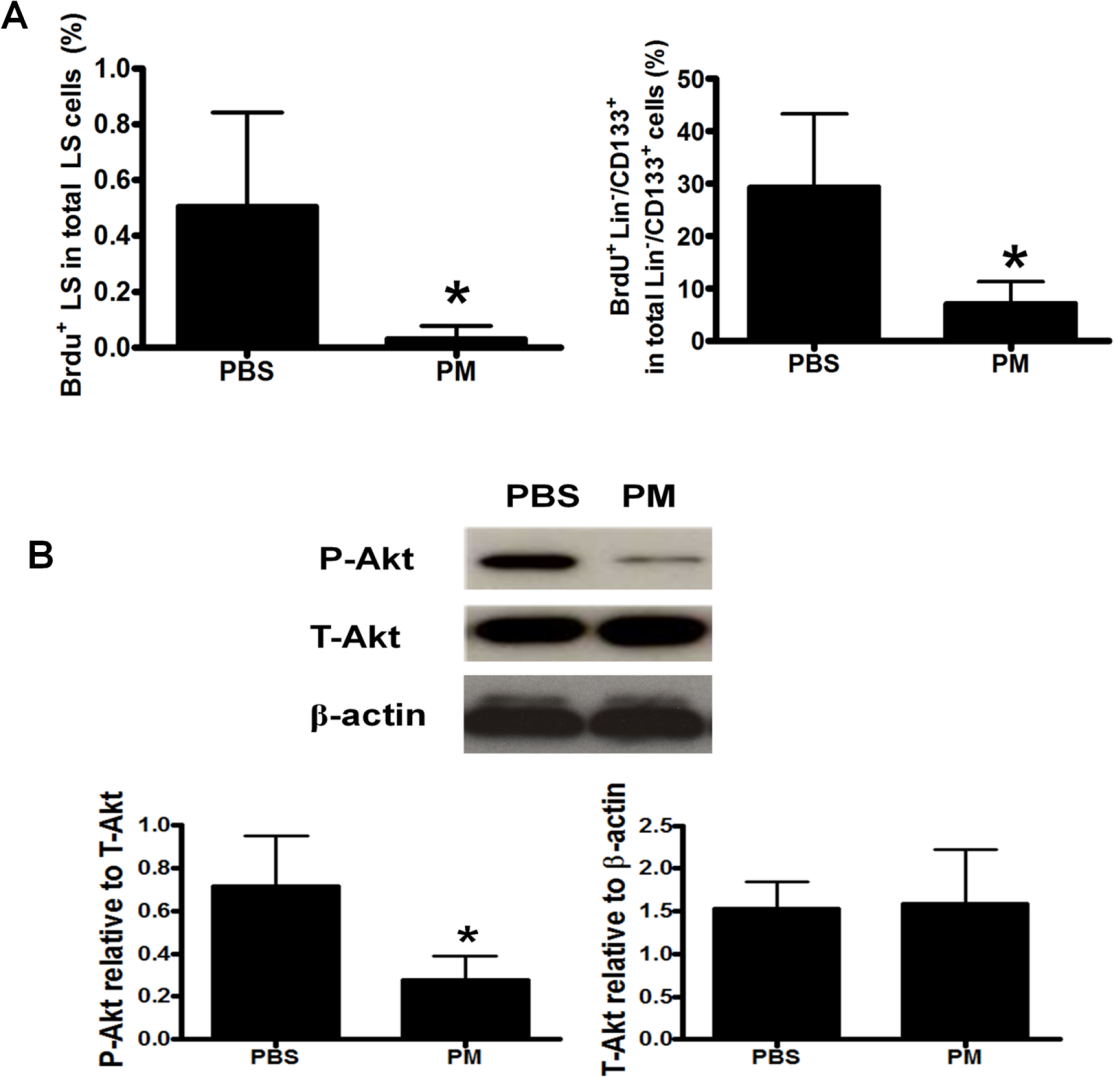
PM exposure reduced in vivo BMSCs proliferation. After exposure with PM or PBS for 1 month, the C57BL/6 mice were injected (i.p.) with 1 mg BrdU 12h before sample collections. After staining with Lineage cocktail Pacific Blue, Sca-1 PE-Cy5 and CD133 PE, cells were permeabilized and stained with anti-BrdU FITC. The *in vivo* BMSCs proliferation rate (A) and p-Akt level (B) were significantly decreased in the mice with PM exposure compared with the PBS control group. * PM vs PBS, P<0.01, n = 8.

To explore the mechanism for decreased BMSCs population by PM exposure, we measured the *in vivo* BMSCs proliferation rate. PM exposure significantly decreased the *in vivo* proliferation rate of LS and Lin^-^/CD133^+^ cells by 5–13 folds over the control (**Fig 2A**). Akt signaling pathway is closely related to the cell proliferation [33]. To illustrate the mechanisms underlying PM-induced reduction of BMSCs population, we observed that the level of P-Akt in the BM cells was substantially decreased by 2.7 folds in the mice exposed to PM compared to the control group (**Fig 2B**).

### PM exposure increased intracellular ROS production in BMSCs

**Fig 3:**
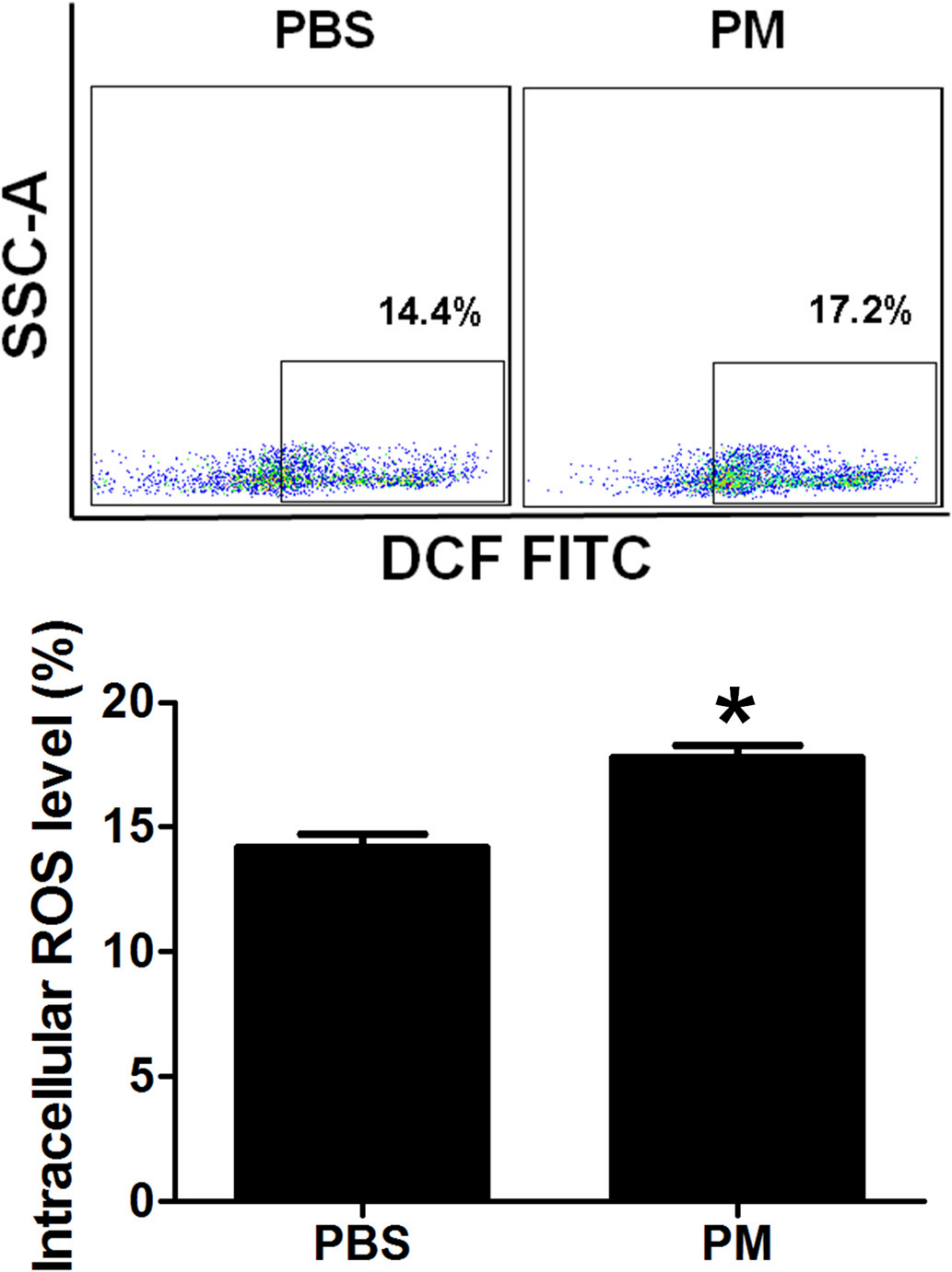
PM exposure significantly increased ROS production in BM cells. Intracellular ROS production was quantitatively determined using the ROS Detection Reagents-FITC in the bone marrow cells. Intracellular ROS formation was significantly increased in the bone marrow in the wild type mice with PM exposure. * PM vs PBS, P<0.001, n = 8.

It was reported that PM exposure could increase ROS production [18, 19]. Thus, we hypothesized that PM-induced ROS production could occur in BMSCs. We measured intracellular ROS production after exposure to PM and observed that intracellular ROS level was indeed significantly increased in the BMSCs in the mice with PM exposure (**Fig 3**).

### NAC treatment or AON overexpression effectively blocked ROS production by PM in BMSCs

**Fig 4:**
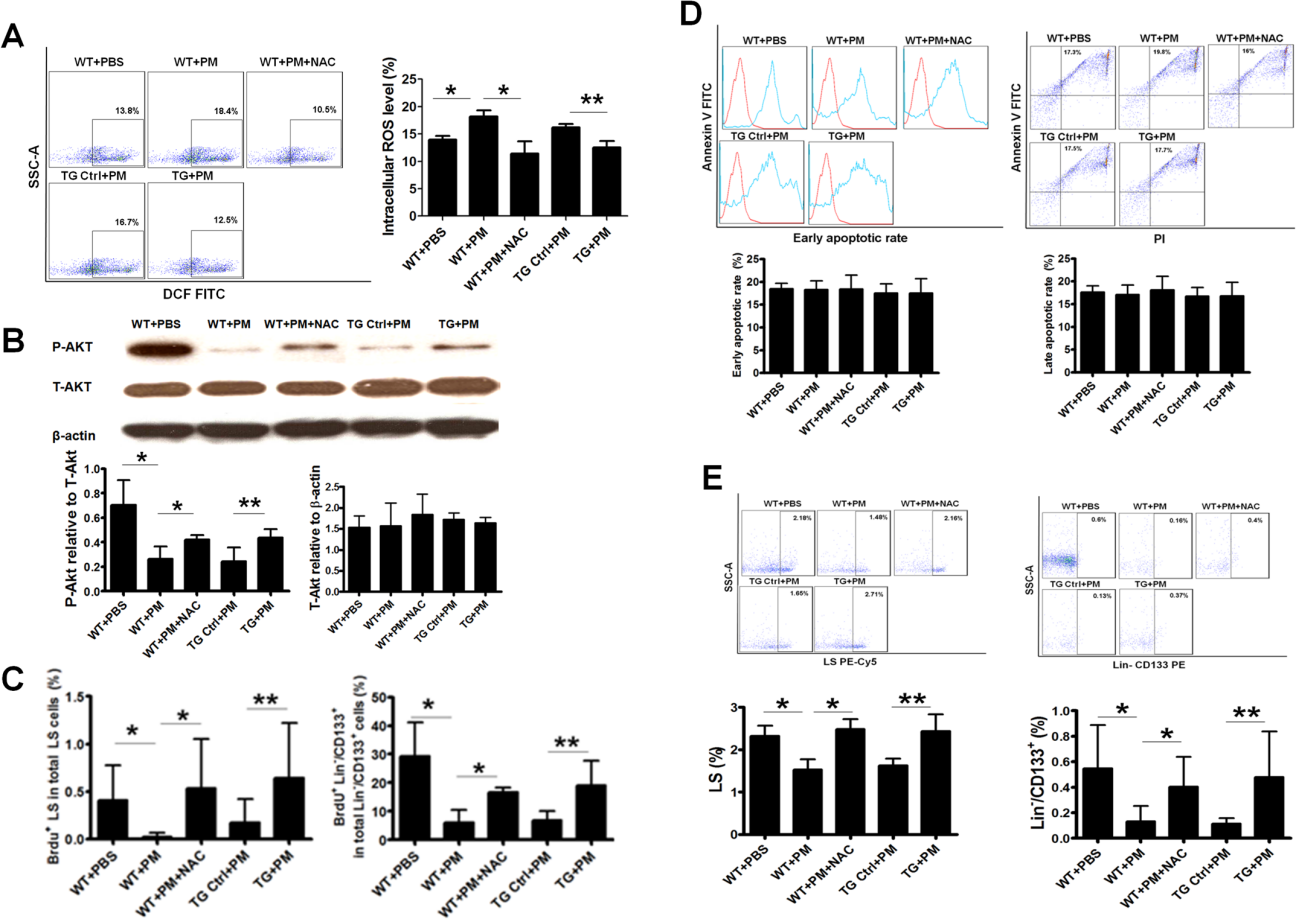
NAC treatment or AON overexpression reversed the detrimental effects of PM exposure on BMSCs. (A) Increased BM intracellular ROS production was completely blocked by NAC treatment or AON in the mice exposed with PM. WT+PBS: C57BL/6 mice with PBS treatment; WT+ PM: C57BL/6 mice with PM exposure; WT+ PM+NAC: C57BL/6 mice with PM exposure and NAC treatment; TG Ctrl+PM: TG mouse littermates with PM exposure; TG+ PM: TG mouse with PM exposure. * WT+ PM or WT+ PM+NAC vs WT+PBS, P<0.01, n = 8; ** TG Ctrl+ PM vs TG+ PM, P<0.05, n = 8. (B) Decreased level of P-Akt was significantly increased by NAC treatment or AON in the mice exposed with PM. * WT+ PM or WT+ PM+NAC vs WT+PBS, P<0.01, n = 8; ** TG Ctrl+ PM vs TG+ PM, P<0.01, n = 8. (C) Both BM LS and Lin^-^/CD133^+^ cell proliferation rate was completely reversed by NAC treatment or AON in the mice exposed with PM. * WT+ PM or WT+ PM+NAC vs WT+PBS, P<0.05, n = 8; ** TG Ctrl+ PM vs TG+ PM, P<0.05, n = 8. (D) Both early apoptotic and late apoptotic rate of BMSCs were not changed in all the mice with different treatments. n = 8. (E) Decreased level of BMSC population was completely restored by NAC treatment or AON in the mice exposed with PM. * WT+ PM or WT+ PM+NAC vs WT+PBS, P<0.01, n = 8; ** TG Ctrl+ PM vs TG+ PM, P<0.01, n = 8.

To determine whether ROS was the cause of PM-induced inhibition of BMSCs proliferation, both pharmacological and transgenic approaches were employed to block ROS generation. The WT mice were co-treated with PM and NAC to inhibit ROS formation. We also used a TG mouse model that over-expressed AON with reduced ROS production. We confirmed that BM intracellular ROS production induced by PM exposure was effectively blocked in NAC-treated mice and in the TG mice overexpressing the AON (**Fig 4A**).

### NAC treatment or AON overexpression partially prevented PM-induced inhibition of Akt phosphorylation

To determine whether the decreased level of P-Akt in the mice exposed to PM was induced by ROS, we measured the total and P-Akt levels in BM cells from NAC-treated and TG mice. P-Akt level was partially, yet significantly recovered in the mice exposed to PM and treated with NAC or over-expressing AON as compared to their controls (**Fig 4B**).

### NAC treatment or AON overexpression effectively reversed PM-induced inhibition of BMSCs proliferation and reduction of BMSCs population

We then determined whether NAC treatment or over-expressing AON could prevent suppression of BMSCs proliferation by PM. As expected, the reduced proliferation rate of both LS and Lin^-^/CD133^+^ cells after PM treatment was significantly reversed in either NAC-treated mice or TG mice following PM exposure (**Fig 4C**) without change in apoptosis (**Fig 4D)**.

Finally, we examined whether inhibition of ROS production could maintain BMSCs population during PM exposure. As shown in **Fig 4E**, the decreased BMSCs population by PM exposure was completely reversed by NAC treatment or AON overexpression. Thus, our data suggested that ROS production induced by PM exposure was indeed a major cause for the decreased population of BMSCs due to inhibition of their *in vivo* proliferation, not induction of apoptosis.

## Discussion

In the present study, we demonstrated that PM exposure significantly decreased the BMSCs population in association with inhibition of Akt phosphorylation. We further demonstrated that ROS production by PM was a major mechanism for decreased BMSCs proliferation and Akt signaling. Treating the mice with antioxidant NAC or overexpression of AON significantly decreased BMSCs intracellular ROS level, partially reversed the suppression of P-Akt expression, effectively reversed the inhibition of BMSCs proliferation rate, and restored the BMSCs population in the mice with PM exposure. To our knowledge, this was the first time to demonstrate that PM exposure decreased the population of BMSCs through inhibition of their proliferation due to ROS-mediated mechanisms (could be partially due to attenuation of Akt signaling) without change in apoptosis.

Air pollution or cigarette smoking had significant impact on the number and function of various SCs including embryonic SCs (ESCs), spermatogonial SCs (SSCs), Clara cells (SCs of the bronchiolar epithelium). CS exhibited cytotoxic action on human ESCs (hESCs) and mouse ESCs (mESCs), induced oxidative stress, apoptosis, and telomere shortening in ESCs, inhibited cell adhesion and growth, and compromised embryo development [34–36]. CS might also induce mutation of SSCs gene and alterations in hESCs gene expression (especially those characteristic for mesoderm and ectoderm development) [37]. Increased expression of Notch, Wnt or TGF-β genes by smoking resulted in retention of the cells in pluripotent state [38]. In addition, acute exposure of mESCs to CS or cadmium could cause immediate cell death, and decrease their pluripotency, while chronic exposure could lead to DNA damage and telomere shortening [39, 40]. Coal dust exposure resulted in the disappearance of proliferating cell nuclear antigen in rat Clara cells [41]. Although cigarette smoking could recruit SCs into lung [42, 43], negative impact including interfering mesenchymal SCs (MSCs) homing by targeting microvascular endothelial cells and differentiation into endometrial cells and blood vessel was reported [15, 16]. The present study showed that PM was able to decrease the number of BMSCs due to reduced proliferation, not increased apoptosis, via ROS-mediated impairment of P-Akt signaling.

ROS and oxidative stress are critically involved in the regulation of stem cell function [44, 45]. ESCs have been shown to develop a transient cell cycle arrest when continuously exposed to a short-term sublethal concentration and duration of H_2_O_2_ without significant changes in their capacity of cell proliferation, self-renewal, and pluripotency [46]. Similarly, a concentration-dependent decrease in cell viability was observed after exposure of rat MSCs to H_2_O_2_ [47]. Moreover, oxidative stress could trigger MSCs cell death *in vitro* in association with enhanced Akt activation and increased secretion of growth factors (VEGF, FGF-2, and IGF-1) was prevented by overexpression of Hsp20 [48]. Furthermore, mice hematopoietic SCs senescence were induced after exposure to a sub-lethal dose of total body irradiation through persistent increase in ROS production *In vivo* [48]. We also demonstrated that ROS formation was important in the action of ox-LDL on BMSCs including inhibition of Oct-4 expression, proliferation, and endothelial differentiation [33]. Interestingly, the differentiation of mature blood cells from hematopoietic progenitors in Drosophila could be promoted with higher ROS level [49]. Particles especially PM widely existed in our environment and could carry ROS within gas phase [18, 19] or water phase (aerosol) [20] into the lower respiratory tract to create an increased risk on health. There is growing evidence for oxidative stress in response to air pollution in different organs [21]. It has been reported that CS and mosquito coil smoke could induce oxidative stress to impair the ESCs and compromise germ cells production and embryo development [36, 50, 51]. We also observed that the decreased proliferation of BMSCs was related to increased ROS production and decreased level of P-Akt *in vitro* [33]. In the present study, our *in vivo* data showed that PM exposure inhibited BMSCs proliferation through ROS-mediated inhibition of Akt signaling.

ROS is an important signaling molecule that is involved in regulaitons of a variety of signaling pathways including Akt pathway as summarized in recent reviews [52, 53]. There are extensive and complex interactions between ROS and Akt pathway in both normal and cancer cells. Tetrandrine inhibits the growth of mouse endothelial cells and induces G1/S arrest through ROS/Akt pathway [54]. The anti-angiogenic activity of magnolol is considered to be due to ROS-mediated suppression of the PI3K/AKT/mTOR signaling pathway in the endothelial-like cells derived from mouse embryonic stem cells [55]. ROS generation and PI3K/Akt signaling play key roles in the survival of sulforaphane-treated human mesothelioma MSTO-211H cells [56]. ROS-mediated PI3K/AKT/mTOR/p70S6K signaling pathways was critical to the effects of cathepsin S on the regulation of autophagy and apoptosis in human glioblastoma cells [57]. In the present study, we observed that PM exposure inhibited BMSCs proliferation via ROS-mediated mechanism(s) (partially through suppression of Akt signaling). It is certainly possible that other pathways might also be affected by PM exposure. Future studies are needed to define the role of other pathways in the effect of PM exposure on BMSCs.

Antioxidant enzyme and antioxidants have been examined for their effects on ROS and stem cells. Supplementation of selenite restores the basal activity of antioxidative selenoenzymes, reduces ROS accumulation in human MSCs, and attenuates oxidative cell damage in BMSCs *in vitro* [58]. Treatment of MSCs with ROS scavenger berberine protects the cells against ROS-induced apoptosis *in vitro* [59]. Prevention of oxidative stress with daily subcutaneous injection of SOD-mimic for 4 weeks significantly decreased the intracellular ROS level in BM mononuclear cells (BM-MNCs) in diabetic mice, and increased the percentage of EPCs and their potency of differentiation into endothelial cells [60]. Treatment of mice with total body irradiation with Mn(III) meso-tetrakis-(N-ethylpyridinium-2-yl) porphyrin (MnTE), a SOD mimetic and potent antioxidant, significantly inhibited the increases in ROS production and DNA damage and cell senescence in HSCs in the BM [61]. Treatment with the anti-oxidant NAC (by scavenging ROS) was able to restore the impaired self-renewal potential and functional activity of HSCs with high ROS level [45]. NAC treatment also protected BMSCs against the toxic effect of low concentration ox-LDL, and restored their endothelial differentiation potential impaired by ox-LDL [33]. Our data showed that NAC or overexpression of AON could completely block BMSCs intracellular ROS production, partially restore P-Akt level, effectively reversed the decreased proliferation rate of BMSCs and increased the BMSCs number to normal level in the mice with PM exposure.

Of course, there are still lots of questions that need to be addressed on PM induced structural and functional impairment on BMSCs. For example, does PM also affect the differentiation potential and how? In the present study, we observed that PM could reduce the number of BMSCs that might serve as the main source of EPCs. This could be one of the reasons for reduced EPCs number in circulation. Antioxidant NAC or overexpression of AON could only partially reverse the impaired P-Akt expression with PM exposure, suggesting that there might be other mechanisms for the regulation of P-Akt signaling during PM exposure. How does ROS regulate the P-Akt signaling? Does PM also trigger autophagy of BMSCs? All these questions require further studies.

## Conclusion

We demonstrated that PM exposure significantly decreased BMSCs population due to reduced *in vivo* proliferation of BMSCs through ROS-mediated mechanism(s), might be partially due to impairment of Akt signaling without change in apoptosis. The antioxidant NAC or overexpression of AON was able to reverse the adverse effect of PM on BMSCs.
